# Establishing the Role of Neurogenic Inflammation in the Pathogenesis of Periodontitis: A Systematic Review

**DOI:** 10.7759/cureus.26889

**Published:** 2022-07-15

**Authors:** Smrithi V Varma, Sheeja Varghese, Vijayashree J Priyadharsini, Jayakrishnan Radhakrishnan, Sajan V Nair

**Affiliations:** 1 Department of Periodontology, Saveetha Dental College, Chennai, IND; 2 Clinical Genetics, Saveetha Dental College, Chennai, IND; 3 Department of Community Oncology, Regional Cancer Center, Kerala, IND; 4 Department of Orthodontics, Sri Sankara Dental College, Kerala, IND

**Keywords:** neuropeptides, systematic review, periodontal disease, periodontitis, neurogenic inflammation

## Abstract

The role of neurogenic inflammation in various systemic diseases has been well established, but there is a dearth of studies and evidence regarding its role in periodontitis. This study aimed to systematically review the evidence in establishing the role of neurogenic inflammation in chronic periodontitis. Databases such as PubMed, Scopus, and Google Scholar were reviewed. We analyzed studies of any design that compared and evaluated the presence of neuropeptides such as substance P, calcitonin gene-related peptide, neurokinin A, neuropeptide Y, and vasoactive intestinal polypeptide in systemically healthy patients with and without periodontitis. We screened 2,495 articles and abstracts electronically and manually, which yielded 191 articles relevant to our study. Full-text examination of these 191 articles led to the final inclusion of 14 publications. Most studies here confirmed an association between various neuropeptides and periodontitis, but there is a high heterogeneity between the studies, making it necessary to clarify the mechanism between these two. Although most studies included in this review found a positive association between neurogenic inflammation and periodontitis, the evidence is of moderate to low quality.

## Introduction and background

Chronic periodontitis is a disease of inflammatory origin caused predominantly by bacteria present in the dental plaque. Although bacteria are a well-established cause of periodontitis, their presence alone is not enough to produce advanced periodontal tissue destruction. Apart from being one of the reasons for tooth loss, periodontal diseases are associated with many systemic diseases in developing and developed nations. A neurogenic component is integral to periodontitis [1]. The role of neurogenic inflammation in various systemic diseases has been well established. Many inflammatory diseases, including periodontitis, have been implied to have a neurogenic component. Jancso and Szolcsany, in 1967, introduced the term neurogenic inflammation [2]. Neurogenic inflammation is a protective mechanism; however, severe or prolonged stimulation might result in injury rather than repair. When a chemical combines with chemical irritant receptors on sensory nerves, it can release Substance P (SP) and other inflammatory neuropeptides resulting in neurogenic inflammation [3]. Neurons generate biologically active peptides known as neuropeptides (i.e., peptide neurotransmitters) [4]. A neuropeptide is a peptide synthesized and released from neurons, and its actions are biologically mediated through extracellular receptors on the target cells [4]. A brief outline of the various neuropeptides and their mode of action are given in Table 1 [2,5-17]. We conducted this study to systematically review the evidence in establishing the role of neurogenic inflammation in chronic periodontitis.

**Table 1:** A brief outline of the different neuropeptides and their modes of action SP, substance P; CGRP, calcitonin gene-related peptide; NKA, neurokinin A; NPY, neuropeptide Y; VIP, vasoactive intestinal polypeptide

Neuropeptide	Origin	Amino Acid Chain	Functions	Location	Mode of Action
SP	SP was initially reported in the 1930s by Von Euler and Gaddum (1931) [6], Chang and Gaddum (1933) [7], and Gaddum and Schild (1934) [8].	11-amino-acid peptide [2]	Vasodilator activity is a prominent feature of SP. According to Brain and Williams (1988) [9], SP can modulate vasodilator activity, suggesting an important functional significance to this co-localization.	SP is in peripheral nerves, including enteric neurons and capsaicin-sensitive primary afferent neurons.	Pro-inflammatory
CGRP	CGRP was initially discovered in 1982 [10].	37-amino-acid peptide	According to Brain et al., 1985, CGRP has potent vasodilator activity frequently co-localized with SP [5].	CGRP is widely distributed throughout the central and peripheral nervous systems.	Anti-inflammatory
NKA	NKA was discovered and characterized in 1983 [17].	10-amino-acid peptide	NKA increases vasodilatation, microvascular permeability, and plasma extravasation.	NKA in peripheral nerves, including enteric neurons and capsaicin-sensitive primary afferent neurons.	Pro-inflammatory
NPY	NPY was initially isolated from the porcine brain (Tatemoto, 1982) [12]	36-amino-acid peptide	NPY has potent vasoconstrictor activity NPY (Lundberg et al., 1985) [13].	NPY is widely distributed throughout the central and peripheral nervous systems.	Pro-inflammatory
VIP	VIP was initially isolated from pig intestinal extracts (Said and Mutt, 1970) [11]	28-amino-acid peptide	VIP is an immunomodulatory peptide (Bellinger et al., 1996) [14] that regulates the production of pro-and anti-inflammatory mediators (Pozo et al., 2000 [15]; Ganea and Delgado, 2002) [16], relaxes smooth muscle and induces salivary secretion.	VIP is located in the central and peripheral nervous systems.	Anti-inflammatory

## Review

We followed the Preferred Reporting Items for Systematic Reviews and Meta-analysis (PRISMA) guidelines to conduct this systematic review [18]. We sought to determine the weight of evidence existing to establish the link between neurogenic inflammation and periodontitis. Our review included studies of any design (e.g., randomized controlled trials, cohort studies, case-control studies, cross-sectional studies) that compared and evaluated the presence of any one of the neuropeptides such as SP, calcitonin gene-related peptide (CGRP), neurokinin A (NKA), neuropeptide Y (NPY), and vasoactive intestinal polypeptide (VIP) in systemically healthy patients with and without periodontitis. The studies included defined periodontal disease according to any clinical periodontal indexes. Our search strategy used a combination of Medical Subject Headings (MeSH) terms as in Table 2. Our review excluded review articles, articles published in languages other than English, studies that lacked baseline data, and animal studies.

**Table 2:** MeSH search terms MeSH, Medical Subject Heading

MeSH Terms	
Adult Periodontitides	Neuropeptide Receptors
adult periodontitis	Neuropeptide Y receptor
Adult Periodontitis	Neuropeptide Y
Adult	Neuropeptide
Aggressive	Parodontoses
Calcitonin Gene-Related Peptide	Parodontosis
Chronic periodontitides	Periodontal attachment loss
chronic periodontitis	Periodontal disease
Chronic	Periodontal
Circumpubertal Periodontitis	Periodontitides
Circumpubertal	Periodontitis
Early Onset Periodontitis	Periodontoses
Early-Onset Periodontitides	Prepubertal Periodontitis
Early-Onset	Prepubertal
Juvenile Periodontitides	Refractory periodontitis
Juvenile Periodontitis	Vasoactive intestinal peptide
Juvenile	-

Search strategy and data extraction

**Figure 1:**
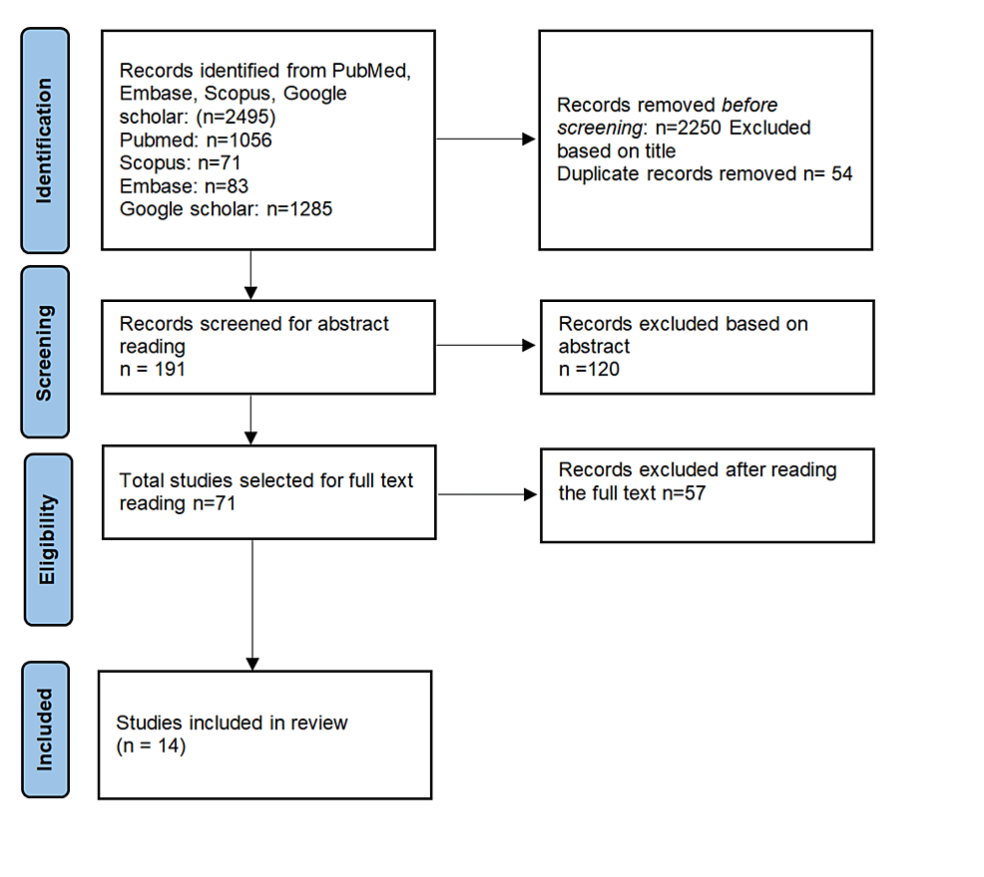
Structural outline of the search strategy (PRISMA flow diagram) PRISMA, Preferred Reporting Items for Systematic Reviews and Meta-Analysis

We reviewed databases such as PubMed, Scopus, and Google Scholar. An independent reviewer searched the reference lists of the original and review articles using the MeSH terms. No year restrictions were applied while searching for articles. Screening 2,495 articles and abstracts both electronically and manually yielded 191 relevant articles. Full-text examination of the 191 eligible articles led to a final inclusion of 14 publications. We assessed the quality and main study characteristics of each article. The variables included adequately defined periodontal disease criteria and whether the article assessed one or more of the neuropeptides. Data from 14 selected articles were obtained and appraised by an independent reviewer. Figure 1 presents a structural outline of the search strategy.

Evaluation process

Studies involved in this systematic review needed to present confounding factors controlled through randomization, matching, stratification, restriction, or statistical modeling to impede the course of periodontal disease. We included studies where confounding factors have been controlled through randomization, matching, and restriction. Studies included had to also state adequate criteria by which periodontal disease is defined (e.g., clinical attachment loss, probing depth, alveolar bone loss, bleeding on probing, gingival recession, and missing teeth). The studies had to have adequate criteria for establishing neurogenic inflammation.

Results

Study Characteristics

Of the 14 studies included, seven were case-control studies, three were longitudinal, two were in vitro, and two were cross-sectional studies. All studies were published in English between 1989 and 2019. The studies were conducted in eight different countries (six in Ireland, one in India, Sweden, Australia, Japan, England, and China, and two in Turkey). The patient sample ranged from eight to 179 patients. Nine studies included healthy subjects and periodontitis patients, three assessed pre- and post-periodontitis treatment, one consisted of only a periodontitis group, and one contained patients with chronic migraine with or without periodontitis.

Study Outcomes and Parameters Assessed

The data were extracted and organized from the included articles by heading, author's name, type of study, study location, sample size, study groups and characteristics, the statistical method used, outcome parameters, employed diagnostic method, results, and the sample collected in each study and conclusions. Tabular columns were created and rechecked to confirm the accuracy of the extracted information. We could not perform a meta-analysis due to the heterogeneity of the studies.

Outlines of the available studies are presented in Table 3 [19-25], Table 4 [26-28], Table 5 [29-30], and Table 6 [31-32]. Several studies analyzed the relationship between periodontal disease and more than one neuropeptide. In all, 11 of 14 studies suggested that periodontal disease is a risk factor for neurogenic inflammation. Five neuropeptides (SP, CGRP, VIP, NKA, and NPY) were assessed to evaluate their role in the pathogenesis of periodontitis. Nine of 14 studies evaluated SP while eight used gingival crevicular fluid (GCF) as the sample.

**Table 3:** Role of neuropeptides in periodontitis case-control studies ANOVA, analysis of variance; M, male; F, female; CI, confidence interval; NA, not applicable; GCF, gingival crevicular fluid; SP, substance P; CGRP, calcitonin gene-related peptide; NKA, neurokinin A; NPY, neuropeptide Y (NPY)

Study	Sample Collected	Statistical Method	Outcome Parameters	Sample Size	Groups	Results	Conclusions
Lundy et al., 2000 [19] Ireland	GCF	NA	SP, CGRP, NKA	Group 1: 3F, 1M	Periodontitis	Extensive degradation of CGRP at 1, 10, and 180 minutes; lowest molecular mass (m/z) identified at 180 minutes was 1177.2 m/z	No metabolism of SP, NKA, or CGRP in healthy tissues. The periodontitis group showed a greater breakdown of CGRP than SP and NKA.
				Group 2: 4F	Healthy		
Lundy et al., 2009 [20] Ireland	GCF	The T-test and Mann-Whitney test	NPY	Group 1: 10F, 10M;	Periodontitis	NPY, 39.8 ng; NPY concentration, 96.4 ng/microL	Significantly elevated NPY in the healthy group. NPY has an anti-inflammatory role.
				Group 2: 10F, 10M	Control	NPY, 161 ng;, NPY concentration: 645.7 ng/microL	
Lundy et al., 1999 [21] Ireland	GCF	Wilcoxon signed-rank test, Friedman two-way ANOVA, Wilcoxon-Mann-Whitney	CGRP	Group 1: 9F, 9M	Periodontitis	Subgroup 1, CGRP, 6.3 pg, Concentration, 698.7 pg/microL	Significantly elevated levels of CGRP-IR in periodontally healthy groups compared to gingivitis and periodontitis.
						Subgroup 2, CGRP, 2.4 pg, Concentration, 19.8 pg/microL	
						Subgroup 3, CGRP, 0 pg	
				Group 2: 14F, 5M	Control	CGRP, 8.5 pg, Concentration 433.8 pg/microL	
Chen et al., 2000 [22] China	Gingival biopsy	Quantimet 970	SP, CGRP	Group 1: 15F, 5M	Moderate chronic periodontitis	Mean optical absorption, SP: 0.127856; CGRP: 0.126876	SP and CGRP exert an inflammatory effect. No intergroup expression changes in response to inflammation.
				Group 2: 15F, 5M	Healthy controls	Mean optical absorption, SP: 0.117973; CGRP: 0.119763	
Sakallioğlu et al., 2008 [23] Turkey	Gingival biopsy	One-way ANOVA, post hoc Turkey test	SP, CGRP	27 Subjects	Group 1: Smokers with periodontitis	Mean SP: 35.35±0.79 pg/ml Mean CGRP: 44.32±0.54 pg/ml	Mean SP and CGRP were higher in Group 1 than in Groups 2, 3, and 4
					Group 2: Smokers with periodontally healthy teeth	Mean SP: 28.81±0.70 pg/ml, Mean CGRP: 40.74±1.00 pg/ml	
					Group 3: Nonsmokers with periodontitis	Mean SP: 32.47±1.39 pg/ml, Mean CGRP: 25.66±2.05 pg/ml	
					Group 4: Nonsmokers with periodontally healthy tooth	Mean SP: 27.91±0.95 pg/ml, Mean CGRP: 40.70±1.21 pg/ml	
Leira et al., 2019 [24] England	Blood samples	Kolmogorov-Smirnov	CGRP	179 Subjects	Group 1: Chronic migraine with periodontitis	Mean serum CGRP: 19.7±6.5 pg/mL (Beta=0.003; 95% CI: 0.001 to 0.006, p=0.031)	Increased periodontal inflammation is associated with higher circulating levels of CGRP in chronic migraine group.
					Group 2: Control group	Serum CGRP: 15.3±6.2 pg/ml	
Sert et al., 2019 [25] Turkey	GCF peri-implant sulcular fluid	Shapiro-Wilk, ANOVA, post hoc Tukey, students t-test, paired-sample t and Kruskal-Wallis, Mann-Whitney U and Wilcoxon	SP, CGRP, NKA, NPY	39 Subjects	Group 1: Healthy group	Mean SP: 31.24±2.83 pg/microL 30s)	A rise in SP and NKA levels and a drop in CGRP and NPY levels in diseased states
						Mean NKA: 67.59±3.00 pg/microL 30s	
						Mean CGRP: 48.99±0.78 pg/microL 30s	
						Mean NPY: 583.11±13.58 pg/microL 30s	
					Group 2: Periodontitis	Mean SP: 137.57±7.02 pg/microL 30s	
						Mean NKA: 109.32±4.61 pg/microL 30s	
						Mean CGRP: 23.92±2.45 pg/microL 30s	
						Mean NPY: 108.33±18.31 pg/microL 30s	

**Table 4:** Role of neuropeptides in periodontitis longitudinal studies GCF, gingival crevicular fluid; SP, substance P; NKA, neurokinin A; VIP, vasoactive intestinal polypeptide; NKA-LI, neurokinin A-like immunoreactivity; SP-LI, Substance P-like immunoreactivity

Study	Sample Collected	Statistical Method	Outcome Parameters	Sample Size	Groups	Results	Conclusions
Linden et al., 2002 [26] Ireland	GCF	Wilcoxon signed-rank test, Mann-Whitney	VIP	20 Subjects	Group 1: Periodontitis	Pretreatment: VIP 302.0 pg; Concentration, 524.3 pg/microL	Increased VIP in periodontitis sites decreased significantly after treatment.
						Post-treatment: VIP 78.0 pg	
					Group 2: Healthy	Pretreatment: VIP 115.5 pg; Concentration, 883.8 pg/microL	
						Post-treatment: VIP 77.8 pg	
Pradeep et al., 2009 [27] India	GCF and Plasma	Kruskal-Wallis	SP	30 Subjects	Group 1: Healthy	GCF: 6.07± 3.43 pg/mL, Plasma: Not detectable	The mean concentration of SP in GCF and plasma was highest in the periodontitis group and significantly declined after treatment.
					Group 2: Gingivitis	GCF: 11.42±4.01 pg/mL, Plasma: 38.80± 2.97 pg/mL	
					Group 3: Periodontitis	GCF: 45.13± 13.99 pg/mL, Plasma: 67.80±11.01 pg/mL	
					Group 4: Post-treatment group	GCF: 7.58±3.25 pg/mL, Plasma: 39.7±3.83 pg/mL	
Lundy et al., 2000 [28] Ireland	GCF	Wilcoxon signed-rank test	SP, NKA	8 Subjects	Group 1: Periodontitis pretreatment	SP-LI, 56.3 pg; Concentration, 140.6 pg/microL	Higher SP-LI and NKA-LI levels in GCF before treatment than after treatment.
						NKA-LI, 30.5 pg; Concentration, 85.4 pg/microL	
					Group 2: Periodontitis post-treatment	SP-LI, 4.2 pg, Concentration, 24.2 pg/microL	
						NKA-LI, 10.6 pg, Concentration, 61.6 pg/microL	

**Table 5:** Role of neuropeptides in periodontitis in in-vitro studies ANOVA, analysis of variance; SP, substance P; CGRP, calcitonin gene-related peptide; NPY, neuropeptide Y; VIP, vasoactive intestinal polypeptide

Study	Sample Collected	Statistical Method	Outcome Parameters	Sample Size	Groups	Conclusions
Luthman et al., 1989 [29] Sweden	Gingival biopsy	NA	SP, CGRP VIP, NPY	33 Subjects	Group 1: Periodontitis	SP, CGRP, NPY, and VIP immune reactivity in periodontitis gingiva but no discernable variations in distribution when compared to clinically healthy sites.
					Group 2: Healthy	
Bartold et al., 1994 [30] Australia	Gingival biopsy	ANOVA	SP	6 subjects	Group 1: Healthy human gingival tissues	SP in the connective tissue between collagenous elements of healthy tissue. SP levels were higher around the blood vessels and inflammatory cell infiltrate of periodontally inflamed tissues.
					Group 2: Inflamed human gingival tissues	

**Table 6:** Role of neuropeptides in periodontitis in cross-sectional and split-mouth studies ANOVA, analysis of variance; SP, substance P; GCF, gingival crevicular fluid; NKA, neurokinin A; NKA-LI, neurokinin A-like immunoreactivity; PPD, periodontal probing depth

Study	Sample Collected	Statistical Method	Outcome Parameters	Sample Size	Groups	Results	Conclusions
Hanioka et al., 2000 [31], Cross-sectional, Japan	GCF	Spearman rank correlation coefficient	SP	48 Subjects	Periodontitis	Significant correlation between SP and PPD (r=0.637, p≤0.001)	An important determinant in GCF is SP which has a significant correlation to PPD.
						Weak correlation between SP and gingival (r=0.177; p=0.23)	
						Weak correlation between SP and plaque index (r=0.008,p=0.96)	
Linden GJ et al., 1997 [32], Split mouth, Ireland	GCF	Wilcoxon signed-rank test, Friedman 2-way ANOVA, Wilcoxon Mann-Whitney	SP, NKA	40 Subjects	Group 1: Periodontitis	Subgroup 1: SP-LI, 10.0 pg; Concentration: 279.6 pg/microL	Greater levels of SPA-LI and NKA-LI A in the periodontitis group than in the control group
						Subgroup 2: SP-LI, 21.1 pg; Concentration: 61.7 pg/microL	
						Subgroup 3: SP-LI, 42.4 pg; Concentration: 43 pg/microL	
					Group 2: Healthy group	SP-LI: 2.0 pg; Concentration: 61.5 pg/microL	

Periodontitis and SP

In Pradeep et al.’s study, the mean concentration of SP was highest among periodontitis patients (GCF: 45.13± 13.99 pg/mL, plasma: 67.80 ± 11.01 pg/mL) and decreases after treatment (GCF: 7.58 ± 3.25 pg/mL, plasma: 39.7 ± 3.83 pg/mL) [27]. This trend was supported by similar studies where pretreatment levels of SP decrease after treatment [19]. However, Luthman et al. found that despite immunoreactivity for SP, CGRP, NPY, and VIP, no discernable differences were observed between healthy and diseased sites [29]. Studies have shown that smokers with periodontitis had higher SP in the gingival biopsy sample than smokers with periodontally healthy teeth, nonsmokers with periodontitis, and nonsmokers with periodontally healthy teeth [23]. Sert et al. had seen similar findings where SP levels were higher in the diseased state than in healthy periodontium [25]. In a cross-sectional study, Hanioka et al. found that SP was significantly correlated to probing pocket depth (r=0.637, p≤.001), therefore, SP could be a key element in GCF [31]. According to Linden et al., a significant increase in SP-like immunoreactivity was seen in subjects with periodontitis (42.4 pg) than in control groups (2.0 pg) [32].

Periodontitis and CGRP

CGRP is a potent vasodilator [5] and is often co-localized with SP. Compared to healthy patients, there was extensive degradation of CGRP in periodontitis patients [19]. A notable increase in CGRP immunoreactivity was detected in periodontally healthy patients compared to those with gingivitis and periodontitis-affected sites [21]. Sert et al. demonstrated that CGRP levels decreased in diseased states [25]. CGRP levels in healthy periodontal tissues were 48.99 ± 0.78 pg/µL 30 s, whereas, in diseased states, they decreased to 23.93 ± 1.80 pg/µL 30s. Some studies revealed higher SP and CGRP levels in smokers with periodontitis than smokers with periodontally healthy teeth or nonsmokers. Leira et al. demonstrated that increased periodontal inflammation was associated with higher circulating levels of CGRP in chronic migraine patients compared to healthy patients [24].

Periodontitis and VIP

VIP is a ubiquitous peptide with a well-established role as an immunomodulatory peptide capable of regulating both pro- and anti-inflammatory mediators. Linden et al. suggested that the amount of VIP decreased significantly after periodontal treatment [26]. VIP levels were 302.0 pg at the diseased site before treatment and 78.0 pg after treatment.

Periodontitis and NKA

Lundy et al. demonstrated that the pretreatment levels of NKA (30.5 pg) decreased after treatment (10.6 pg) [20]. Linden et al. showed a significant increase in NKA in periodontitis-affected subjects compared to healthy controls [31]. However, Sert et al. reported that NKA levels decreased in diseased states [25]. NKA levels in healthy periodontal tissues were 583.11 ± 13.58 pg/µL 30 s but decreased to 108.33 ± 18.31 pg/µL 30s in disease states.

Periodontitis and NPY

NPY is widespread throughout the central and peripheral nervous systems. It is a potent vasoconstrictor and is predominantly anti-inflammatory, as supported by Lundy et al.’s results [20]. Elevated NPY levels are present in the GCF of healthy tissues (161 ng) compared to periodontitis-affected tissues (39.8 ng). Sert et al. found that NPY levels decreased in diseased states [25].

Discussion

This study was an attempt to systematically review the existing data from selected studies on the influence of neurogenic inflammation on periodontitis. This study is the first to systematically review the link between various neuropeptides and periodontitis. Specific neuropeptides could systemically modulate inflammatory disorders such as periodontitis and other orofacial conditions. Tissue samples from gingivitis and periodontitis-affected sites revealed the presence of neurochemical markers, indicating the appearance of neuropeptide in the etiopathogenesis of periodontitis [29-30]. The initiation and progression of periodontal disease are multifactorial, and variations in neuropeptide levels are only part of the neurogenic aspect of the etiology, making it challenging to understand the development of the disease.

Most of the studies included in the present review suggest that periodontitis is associated with neurogenic inflammation. This relationship was derived mainly from case-control, longitudinal, in vitro, and cross-sectional studies. Case-control, longitudinal, and cross-sectional studies supply compelling evidence for this correlation.

Four of the seven studies with CGRP as an outcome parameter showed greater CGRP levels in periodontally healthy sites than in periodontally affected sites [19,21,23,25]. This could be because CGRP inhibits lymphocytic proliferation [33] and interleukin (IL)-2 production [34], inhibiting osteoclastic bone resorption and stimulating osteogenesis. Another explanation could be the rapid breakdown of CGRP due to carboxypeptidase activity in the gingival crevicular fluid obtained from periodontitis sites. This is significant because this neuropeptide can inhibit osteoclastic bone resorption and stimulate osteogenesis [19]. On the contrary, however, two studies reported no discernable differences between the levels of CGRP and SP in periodontitis-affected sites and clinically healthy sites [22,29]. A significant body of evidence studying the relation of SP to periodontitis has been included in this review. SP was higher in periodontitis sites than in clinically healthy sites [19,23,25,27-28,32]. Pro-inflammatory neuropeptides, such as SP, stimulate lymphocyte proliferation [35] and potentiate IL-2 production [36]. SP and NKA are not susceptible to carboxypeptidase activity, so they remain elevated in periodontitis [19]. Many studies have shown a positive association between SP and NKA clinical measurement, demonstrating their effects on periodontal disease severity. However, anti-inflammatory neuropeptides, such as NPY, have an important role in maintaining periodontal health [25].

The cause of increased SP in periodontitis is multifactorial, but one reason is that SP increases the activity of osteoclasts, thus stimulating bone resorption [37]. SP-like immunoreactivity prior to the presence of neutrophil ingress suggests its involvement in the early stages of the inflammatory response [38]. SP could influence leukocyte infiltration through several mechanisms, which is an essential feature of the inflammatory process [28]. Clinical studies showed a significant reduction in pro-inflammatory neuropeptides, such as SP, after periodontal treatment [28-27].

Several studies reported a notable reduction in VIP after periodontal therapy. However, increased VIP levels (as seen in clinically healthy sites) could be due to its anti-inflammatory role. VIP is a macrophage deactivating factor that prevents excessive production of pro-inflammatory factors [26] and inhibits lipopolysaccharide (LPS)-induced tumor necrosis factor-alpha, interleukin-6 (IL-6), and IL-12 production in activated macrophages [39-40]. Down-modulation of CD14 stimulates the production of IL-10, a potent anti-inflammatory cytokine [41]. A significant increase in VIP in periodontitis sites could be because LPS is a potent stimulus for inducing VIP production and secretion in vitro [42]. This shows that SP and VIP have antagonistic roles in periodontal inflammation. An integral part of the host immune response is the maintenance of equilibrium between pro- and anti-inflammatory neuropeptides. Linden et al. found a significant increase in SP-like immunoreactivity and NKA-like immunoreactivity in gingivitis and periodontitis sites as compared to healthy sites [32].

Most studies here confirmed an association between various neuropeptides and periodontitis, but there is a high heterogeneity between the studies, making it necessary to clarify the mechanism between these two. The pathophysiological mechanisms are still unclear, and further studies that use a standardized periodontitis assessment are needed in this regard. Since periodontitis is a multifactorial disease, multiple factors could have influenced the initiation and progression of the disease such as systemic disease, obesity, smoking, poor oral hygiene, stress, and genetics. These factors could have increased the host’s susceptibility to periodontitis. Therefore, ignoring these factors could have influenced the results of this review. All the studies included in this review used different methodological aspects, such as selection guidelines for patients in each group, methods used to evaluate periodontal disease, external variables, such as the population studied, and demographics; this heterogeneity is evident given the difference in the clinical parameters analyzed in each study.

The inconsistencies in how periodontal disease was defined across these studies indicate the presence of potential biases. Clinical attachment level and probing pocket depth are the most used methods of assessing periodontal disease as established in 1959 [43]. Different criteria have been used to define periodontal disease, which could lead to different results given that there are no universally accepted standards for periodontal disease diagnosis. For the studies to be standardized and comparable, there needs to be a universal definition for periodontal disease. Secondly, the insufficient sample size was one concern, increasing the probability of an association observed either by chance or by lack of statistical power. Larger sample sizes can minimize the effect of such bias.

Further limitations of the study would be a lack of information regarding comorbidities, missing data, a limited number of studies, inadequate methodology, and heterogeneity of the underlying condition. Controlling the confounding factors could be challenging, especially for highly prevalent conditions such as periodontitis. The methodical strategy used in these 14 studies is quite different; these differences may account for the conflicting results between studies.

Emerging evidence suggests a strong relationship between the extent and severity of periodontitis and neurogenic inflammation, but this relationship is unlikely to be causal. There is enough evidence to support that both these conditions could have resulted from an imbalance in the pro-inflammatory and anti-inflammatory cytokines.

## Conclusions

Periodontitis is pathophysiologically complex and multifactorial. Many factors are responsible for the initiation and progression of the disease, one of which is neuropeptides. Although most of the studies included in this review found a positive association between neurogenic inflammation and periodontitis, the supporting evidence is moderate to low quality. There are limited numbers of studies on this topic. Therefore, more studies are needed in the future to assess the definitive role of the neurogenic mechanism in the pathophysiology of periodontitis. Future investigations should be directed toward research studies with long-term follow-up periods and better control of confounders; these parameters could further our understanding of the role of neuropeptides in periodontitis.
